# Continuous glucose monitoring combined with artificial intelligence: redefining the pathway for prediabetes management

**DOI:** 10.3389/fendo.2025.1571362

**Published:** 2025-05-26

**Authors:** Chenyang Ji, Tong Jiang, Luolin Liu, Jiale Zhang, Liangzhen You

**Affiliations:** ^1^ Faculty of Medicine, University of Malaya, Kuala Lumpur, Malaysia; ^2^ Key Laboratory of Chinese Internal Medicine of Ministry of Education, Dongzhimen Hospital, Beijing University of Chinese Medicine, Beijing, China; ^3^ Heilongjiang Academy of Chinese Medical Sciences, Harbin, China; ^4^ China Science and Technology Development Center for Chinese Medicine, Beijing, China; ^5^ Institute of Basic Theory for Chinese Medicine, China Academy of Chinese Medical Sciences, Beijing, China

**Keywords:** continuous glucose monitoring, prediabetes, artificial intelligence, type 2 diabetes mellitus, fasting blood glucose

## Abstract

Prediabetes represents an early stage of glucose metabolism disorder with significant public health implications. Although traditional lifestyle interventions have demonstrated some efficacy in preventing the progression to type 2 diabetes, their limitations—such as lack of personalization, restricted real-time monitoring, and delayed intervention—are increasingly apparent. This article systematically explores the potential applications of continuous glucose monitoring (CGM) technology combined with artificial intelligence (AI) in the management of prediabetes. CGM provides real-time and dynamic glucose monitoring, addressing the shortcomings of conventional methods, while AI enhances the clinical utility of CGM data through deep learning and advanced data analysis. This review examines the advantages of integrating CGM and AI from three perspectives: precise diagnosis, personalized intervention, and decision support. Additionally, it highlights the unique roles of this integration in remote monitoring, shared decision-making, and patient empowerment. The article further discusses challenges related to data management, algorithm optimization, ethical considerations, and future directions for this technological integration. It proposes fostering multidisciplinary collaboration to promote the application of these innovations in diabetes management, aiming to deliver a more precise and efficient health management model for individuals with prediabetes.

## 1 Introduction

Prediabetes is characterized by blood glucose levels higher than normal but not high enough to be diagnosed as diabetes. It represents a serious health concern as it significantly increases the risk of developing type 2 diabetes, heart disease, and stroke (1). As a critical early warning sign, prediabetes underscores the need to actively explore and implement effective prevention and control strategies (2).

A glycated hemoglobin (A1C) level between 5.7% and 6.4% indicates prediabetes, reflecting the average blood glucose levels over the past 2–3 months, according to the diagnostic criteria set by the National Institute of Diabetes and Digestive and Kidney Diseases and the American Diabetes Association (ADA) for 2025 (3), this range is recognized as the standard for diagnosing prediabetes. It is important to note that any diagnosis of diabetes must be confirmed through a second test, unless obvious symptoms of diabetes are present. However, A1C should not be used to diagnose type 1 diabetes, and certain medical conditions may lead to false results. Additionally, a fasting plasma glucose (FPG) level between 100 and 125 mg/dL (5.6–6.9 mmol/L) also indicates prediabetes, with the test conducted after an overnight fast (4, 5). In the oral glucose tolerance test (OGTT), a 2-hour blood glucose level between 140 and 199 mg/dL (7.8–11.0 mmol/L) also indicates prediabetes. This test evaluates blood glucose levels after consuming a sugary beverage. Multiple diagnostic criteria and consensus guidelines emphasize the critical role of early detection and intervention in preventing the progression to diabetes. Currently, prediabetes has become a global health issue, with its prevalence continuously rising. However, due to variations in diagnostic standards and data collection methods, accurate global estimates remain challenging (6, 7). Recent studies have shown that the number of people affected globally is steadily increasing, primarily driven by factors such as population aging, unhealthy diets, sedentary lifestyles, and rising rates of overweight and obesity (8, 9). Traditional management of prediabetes typically includes lifestyle interventions, which are most effective (10), such as dietary modifications and exercise adjustments, and sometimes includes pharmacological treatments (11). These compelling pieces of evidence highlight the urgency of managing prediabetes. However, these methods still have limitations, such as a lack of personalization, difficulty in addressing patients’ behavioral and psychological issues, insufficient monitoring, and delayed interventions (12, 13). To overcome these limitations, innovative approaches are crucial, particularly personalized strategies, advanced technologies, and data-driven insights (14). The combination of CGM and AI technology offers a promising solution for the management and prevention of prediabetes, as confirmed by the latest retrospective cohort study (15). Several recent systematic reviews have examined the use of AI in diabetes or CGM applications (16, 17). However, these reviews have primarily focused on type 1 or type 2 diabetes management, algorithm development, or glucose prediction accuracy. In contrast, this article emphasizes the application of CGM-AI integration specifically in the context of prediabetes, a stage often underrepresented in current literature. Furthermore, this perspective highlights implementation strategies, patient empowerment, and health system integration, offering a broader view beyond algorithmic performance. In this approach, artificial intelligence-integrated CGM system, as a transformative innovation tool, provides unprecedented continuous glucose monitoring and drives personalized therapeutic interventions through data analysis, with precision even exceeding that of traditional primary indicators, thereby optimizing strategies for management and prevention of prediabetes (18). This article is a perspective based on an extensive analysis of the current literature and emerging developments related to the integration of CGM and AI in prediabetes management. Given the nature of a perspective article, no formal systematic methodology or PRISMA flowchart is included. Although every effort was made to ensure a comprehensive and balanced discussion, potential limitations inherent in selective literature exploration, such as subjective interpretation and coverage bias, should be acknowledged. In the following sections, this article first discusses the advantages of integrating CGM and AI in prediabetes management, focusing on precision diagnosis, personalized intervention, and decision support. It then elaborates on the AI-enabled health process strategies involving personalized management plans, remote monitoring, and shared decision-making. Subsequently, the challenges and future directions of CGM-AI integration are analyzed. Finally, the review concludes by summarizing key findings and proposing future research directions.

## 2 Precision perspective: the advantages of CGM combined with AI

**Figure 1:**
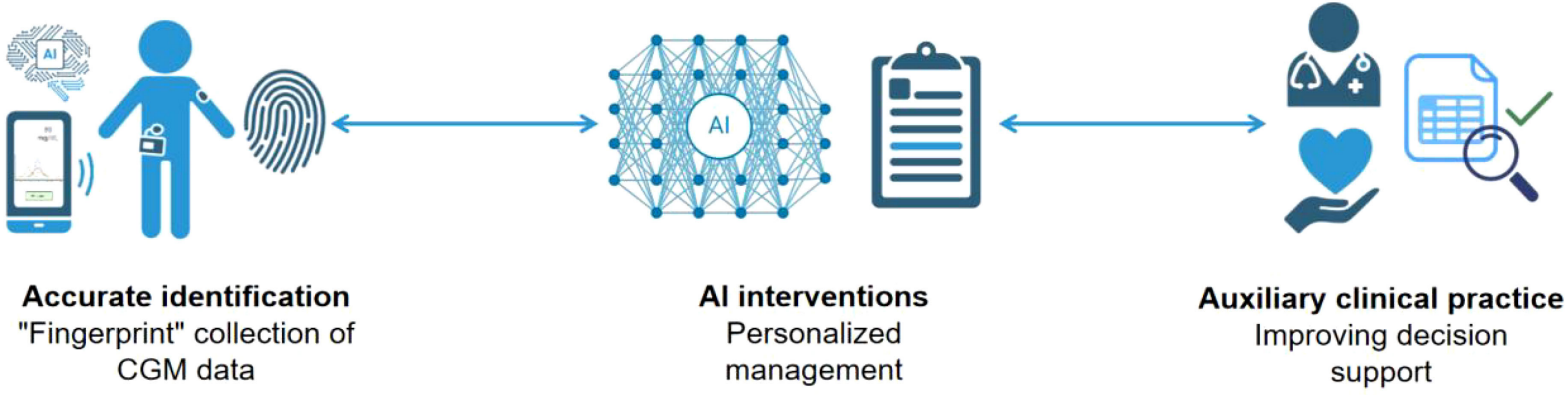
The figure illustrates the processes of “Accurate Identification ‘Fingerprint’ Collection of CGM Data,” “AI Interventions for Personalized Management,” and “Auxiliary Clinical Practice for Improving Decision Support.”. This diagram describes how CGM technology precisely collects patients’ blood glucose data, forming a unique “fingerprint” dataset that lays a solid foundation for subsequent analysis and management. These data are then input into advanced AI systems, where deep learning and analysis of large datasets enable the identification of potential blood glucose patterns, providing scientific evidence for personalized management strategies. This innovative, interconnected approach aims to offer patients a more personalized, efficient, and comprehensive healthcare management plan through accurate data collection, intelligent analytical interventions, and enhanced clinical decision-making support.

The application of AI in prediabetes management is both feasible and desirable, as it facilitates the development of efficient data processing and management tools and devices. The combination of CGM and AI can influence and improve three key areas of prediabetes: diagnosis, management, and treatment(**Figure 1**) (16, 19–21).

### 2.1 Diagnosis: the combination of CGM and AI enhances the accuracy of early detection

Studies have confirmed that even a slight increase in fasting plasma glucose (FPG) levels is clinically significant (22, 23). Specifically, when FPG levels exceed 4.9 mmol/L, insulin resistance in individuals significantly intensifies, directly doubling the risk of developing diabetes. This underscores the importance of maintaining a specific balance to prevent disease progression, with the identification of this critical range being a central element of early intervention (24). Traditional diagnostic methods, such as fasting blood glucose (FBG) and the oral glucose tolerance test (OGTT), while widely used, have limitations in detecting glucose variability and early prediabetes. For example, FBG is subject to individual variability, which can affect its accuracy in identifying early-stage prediabetes (25). The emergence of CGM technology provides a revolutionary solution to this challenge. CGM allows for real-time, continuous monitoring of blood glucose levels, revealing glucose fluctuation patterns that traditional tests are unable to capture, thus improving the accuracy of monitoring and detection (26, 27).

When combined with AI, particularly machine learning and deep learning technologies, the potential of CGM data is further enhanced (28, 29). Emerging research integrating AI-driven diabetes care with cardiovascular risk prediction highlights the interplay between metabolic and cardiac health, reinforcing the value of interdisciplinary approaches (30). By utilizing deep neural networks (DNNs) and explainable AI methods, multiple factors (e.g., pre-meal glucose, insulin dose, nutritional content) can be analyzed to accurately predict postprandial glucose levels (31). Recent studies have developed AI algorithms specifically for meal detection from CGM readings, highlighting subtle patterns not easily detectable by conventional methods. AI can extract complex features from the vast amount of CGM data, identifying subtle patterns associated with the risk of developing type 2 diabetes (T2D) (30). For example, by training deep learning models to predict blood glucose levels and simulate glucose dynamics, AI can assist physicians in more accurately identifying high-risk individuals, even providing early warnings before abnormal blood glucose levels appear (16, 20). By evaluating blood glucose indicators over selected time periods (24 hours, daytime, nighttime, breakfast, lunch, and dinner), “features” are generated to calculate an individual’s window period, along with corresponding sensitivity and specificity ranges. Regarding the reliability of AI methods referenced in this article, the majority are supported by empirical evaluations using large-scale, multi-institutional datasets or validated through cross-validation and real-world clinical trials. For instance, models developed by Woldaregay et al. and Annuzzi et al. were tested on continuous glucose monitoring data under diverse dietary, lifestyle, and insulin dosing conditions, showing high predictive accuracy and generalizability (31, 32). Additionally, many recent frameworks utilize explainable AI (XAI) techniques, which enhance model interpretability and clinician trust. These evidences strengthen the robustness of AI findings and their applicability to prediabetes management. This process enables accurate collection of the “fingerprint” of CGM data, effectively serving as a form of identity recognition (33). This early diagnostic strategy based on CGM and AI not only enhances diagnostic accuracy but also enables personalized assessment of diabetes risk, providing a technological foundation to ensure precision in identification.

### 2.2 Management: the advantages of personalized interventions

Once prediabetes or early T2D patients are identified through CGM and AI technology, the next step is to develop a personalized management plan. The real-time blood glucose data provided by CGM, combined with AI’s data analysis capabilities, allows physicians to gain deep insights into the patient’s glucose fluctuations, including intra-day variability and long-term trends, this approach could enhance precise monitoring of diabetic symptoms (34, 35). Multiple studies have shown that personalized postprandial-targeting (PPT) diets have a more positive impact on blood glucose control and metabolic health in prediabetes compared to the Mediterranean (MED) diet (36), studies examining the associations of related biomarkers, such as glycated hemoglobin (HbA1c), high-density lipoprotein cholesterol (HDL-C), and triglycerides, with cardiac metabolic markers further support this finding (37). The use of CGM and intermittent scanning CGM (isCGM) systems for exercise-related glucose management in type 1 diabetes has been endorsed by position statements from the European Association for the Study of Diabetes (EASD), the International Society for Pediatric and Adolescent Diabetes (ISPAD), as well as the ADA (38). This information is crucial for developing targeted dietary adjustments, exercise plans, and pharmacological treatment strategies.

AI algorithms can also predict the effectiveness of different interventions based on a patient’s specific characteristics, such as age, gender, weight, and lifestyle, thereby recommending the most optimized management strategies (39). This personalized management approach not only improves the effectiveness of treatment but also enhances patient compliance and satisfaction, as the treatment plans are tailored to their specific needs and preferences. Additionally, AI technology has driven the development of CGM devices, such as the Eversense CGM system, which can measure blood glucose levels without traditional invasive needles and provide up to 90 days of real-time glucose monitoring data, significantly improving the convenience and comfort of blood glucose monitoring for patients (40).

### 2.3 Clinical support: enhancing decision-making assistance

In clinical practice, the combination of CGM and AI provides physicians with powerful decision support, significantly enhancing the efficiency and effectiveness of diabetes management. By continuously monitoring blood glucose levels and intelligently analyzing data, physicians can respond rapidly to glucose fluctuations, making timely adjustments to treatment plans and effectively preventing complications. This integration not only enables physicians to identify and manage risk factors that could lead to disease deterioration, such as nocturnal hypoglycemia or postprandial hyperglycemia, allowing for prompt preventive measures, but also improves patient understanding and engagement in their health status (41). Patients can access real-time blood glucose data through mobile applications, monitor glucose trends, and receive AI-based personalized recommendations. This enhancement of self-management capabilities is a crucial component of precision medicine, fostering effective communication between patients and healthcare providers (42).

Additionally, CGM itself, as a key tool for clinical decision support, offers several significant advantages. The CGM system provides real-time, continuous blood glucose readings, dynamically displaying glucose fluctuations throughout the day, offering a more comprehensive and accurate representation of blood glucose compared to traditional self-monitoring of blood glucose (SMBG) (43). CGM can also capture detailed glucose patterns, including trends, variability, and time spent within different glucose ranges. This data helps healthcare providers identify important patterns that SMBG may miss, such as nocturnal hypoglycemia or postprandial glucose spikes (44). By integrating patient characteristics and other clinical information, CGM data can also provide personalized insights into glucose responses, guiding treatment decisions and enhancing patients’ autonomy in diabetes management. Research has shown that the use of CGM significantly improves blood glucose control, particularly in type 1 diabetes patients, while also enhancing patient engagement and increasing adherence to treatment plans (44). Furthermore, CGM helps in the early detection of potential issues, such as hypoglycemia or hyperglycemia, allowing for timely intervention to prevent severe complications. It also enables remote monitoring and telemedicine services, thereby improving healthcare accessibility (45).

## 3 Health process strategy—AI-enabled CGM management

**Figure 2:**
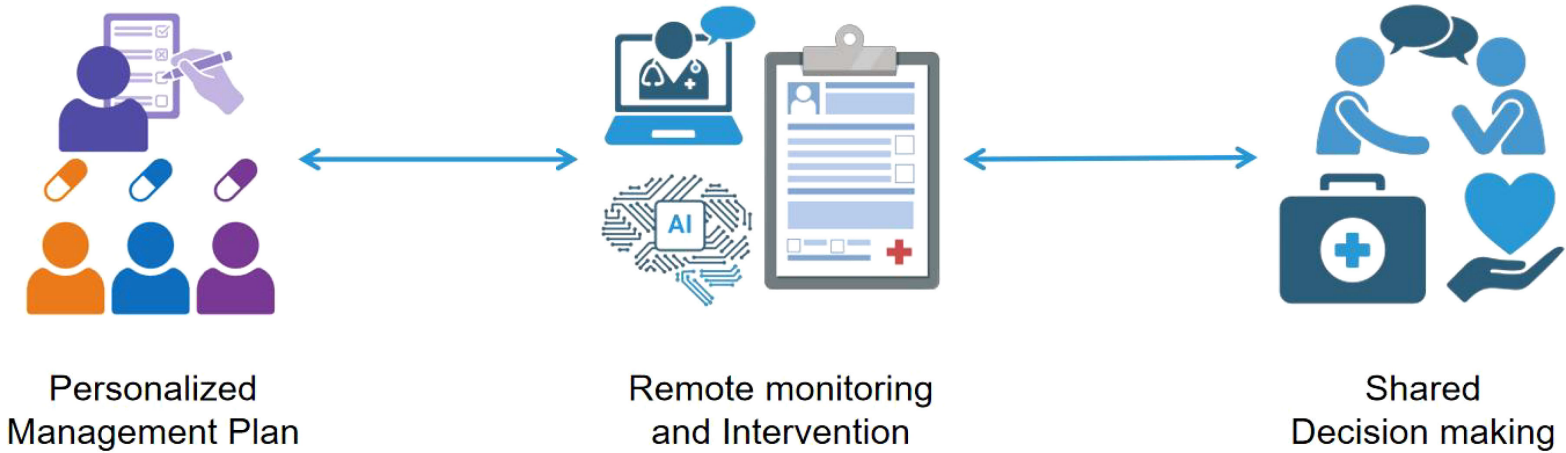
The diagram demonstrates the innovative integration and application of Personalized Management Plan, Remote Monitoring, and Intervention Shared Decision Making in modern healthcare management. At its core, the diagram presents the Personalized Management Plan, which is formulated through comprehensive analysis based on patients’ specific health conditions, lifestyles, and preferences, resulting in a tailored treatment plan. Following this, the introduction of Remote Monitoring and Intervention technology enables the continuous and real-time collection of data, allowing doctors to promptly grasp changes in patients’ health statuses and, when necessary, conduct remote interventions to adjust treatment plans. Lastly, Shared Decision Making serves as the culmination of this innovative integration, emphasizing equal communication and joint decision-making between doctors and patients. Based on the Personalized Management Plan and remote monitoring data, doctors and patients collaboratively discuss the treatment plan, ensuring patients fully understand and participate in their own health management. This decision-making model not only enhances patients’ adherence to treatment but also fosters trust and cooperation between doctors and patients.

With the rapid development of AI technology, its application in the field of CGM is progressively deepening, bringing about revolutionary changes in diabetes management. AI-powered CGM management not only significantly enhances the accuracy and real-time nature of blood glucose monitoring but also plays a crucial role throughout the entire diabetes health management process. Through intelligent data analysis and personalized management strategies, AI is gradually optimizing the health management process for diabetes patients (**Figure 2**).

### 3.1 Personalized management plan: tailored care

In prediabetes management, precision medicine is gradually transitioning from theory to practice, offering patients more personalized, efficient, and compassionate healthcare services. At the core is the development of personalized management plans, which, through CGM and deep insights from AI, tailor dietary, exercise, and medication interventions to individual patients. This data-driven, personalized approach not only overcomes the limitations of the traditional “one-size-fits-all” model but also incorporates the patient’s physiological data, lifestyle, and personal preferences, significantly improving the precision and effectiveness of interventions. For example, AI can analyze CGM data to identify specific foods that cause blood glucose fluctuations and provide optimized dietary recommendations (46). Additionally, by utilizing deep neural networks (DNN) and explainable artificial intelligence (XAI) methods, multiple factors, including pre-meal blood glucose, insulin dosage, and dietary nutritional components, can be analyzed to accurately predict postprandial blood glucose levels in type 1 diabetes (T1DM) patients. This enables the identification of specific foods that influence blood glucose fluctuations and provides patients with optimized dietary recommendations based on personalized data (31); It can also integrate the patient’s activity level to recommend suitable types of exercise, intensity, and duration, helping patients incorporate physical activity into their daily lives. In terms of pharmacological interventions, AI can precisely identify patients suitable for medication treatment and design personalized medication regimens. These personalized management plans, by enhancing patient engagement and adherence, reduce the risk of progression to type 2 diabetes (47). If combined with large language models (LLMs), the problem solving of CGM data can also be optimized to better serve the personalized management plan of patients (48).

### 3.2 Remote monitoring and intervention: cloud-based approach

In the field of telemedicine, the integration of AI and CGM as an innovative cloud-based technology solution demonstrates a unique ability to identify early risks of T2D. This technology can precisely capture postprandial hyperglycemia and the dawn phenomenon, a subtle rise in blood glucose levels during the night—two common glucose fluctuation patterns in prediabetes patients that indicate an increased risk of developing T2D (49, 50). Leveraging cloud-based remote monitoring, CGM not only enables real-time identification of these critical indicators but also provides medical teams with the opportunity for rapid response and the development of personalized intervention strategies, effectively reducing the risk of disease progression.

Additionally, the integration of CGM and AI technologies has opened new avenues for managing prediabetes. The real-time glucose data provided by CGM enables healthcare teams to continuously monitor patients’ blood glucose dynamics and implement timely interventions. AI deeply analyzes this data, automatically generating feedback that includes glucose anomaly alerts, personalized lifestyle recommendations (such as diet and exercise), and medication guidance. Cloud-based services further enhance the effectiveness and accessibility of remote healthcare by securely storing and analyzing patient data, overcoming geographic barriers, and offering timely, efficient medical services to patients in remote areas or those with limited mobility. This approach also reduces unnecessary in-person visits and hospitalizations, effectively controlling healthcare costs (51).

### 3.3 Shared decision-making: enhancing patient participation and empowerment

In the management of prediabetes, patient engagement is crucial to achieving the goals of precision medicine. Shared decision-making, as a patient-centered care model, not only enhances patients’ sense of responsibility for their health management but also improves clinical outcomes through personalized intervention strategies (52). The integration of CGM and AI provides a more robust data foundation for shared decision-making. With real-time monitoring and dynamic feedback, CGM technology allows patients to intuitively understand the relationship between their glucose fluctuations and lifestyle factors such as diet and exercise, while AI algorithms leverage big data analysis to offer optimized management recommendations for both patients and clinicians.

This data-driven shared decision-making process not only enhances patients’ health literacy but also helps them develop personalized intervention plans under the guidance of clinicians, ultimately improving treatment adherence and long-term outcomes. Research indicates that digital health tools contribute to shared decision-making and have a positive impact on improving diabetes care and quality of life, recent studies specifically confirm that real-time CGM-AI feedback significantly enhances patient satisfaction and quality of life by alleviating anxiety related to glycemic fluctuations (53). Moreover, AI technology can further support patients’ proactive interventions by identifying potential health risks. This shift from a “prescriptive” model, reliant solely on healthcare providers, to a “collaborative” model marks a new era in prediabetes management, one that is more precise and efficient. Furthermore, AI technology can further support patients’ proactive intervention capabilities by identifying potential health risks. This shift from a purely “directive” model, which relies solely on healthcare providers, to a “collaborative” model marks the advent of a more precise and efficient era in prediabetes management.

### 3.4 User training and education

One critical component of successfully implementing CGM-AI is the development of tailored training programs for diverse stakeholders. For healthcare providers, this involves competency-based programs to interpret AI-generated insights (e.g., glucose trend predictions) and integrate them into clinical workflows (54). For patients, in contrast, simplified education on data interpretation and self-management tools is required, such as interactive mobile applications that translate CGM data into actionable recommendations (55, 56). For community health workers, who often serve as frontline providers in resource-constrained settings, training should focus on basic device operation and telemedicine coordination to support remote monitoring.

## 4 Challenges and future directions

The use of wearable devices for monitoring physical activity is expected to increase more than fivefold within the next five years, with the integration of medicine and engineering being a key trend in the future of healthcare (57). In recent years, the deep integration of CGM and AI has sparked profound changes in diabetes management, driving a revolution in the healthcare industry. At the heart of this transformation, CGM sensors have gradually become an indispensable revolutionary tool in the field of diabetes treatment. As guardians of blood glucose, they provide precise real-time blood glucose data around the clock, offering us a deeper understanding of blood glucose fluctuations (43). These high-tech devices, which combine wearable, non-invasive, or minimally invasive designs, have not only greatly improved patients’ quality of life but also provided healthcare professionals with unprecedented insights. This allows them to precisely understand the patterns of individual blood glucose fluctuations, enabling the development of more accurate and personalized management plans, significantly promoting the control and alleviation of diabetes.

At the same time, the rapid development of AI in the healthcare field has opened up new possibilities for the long-term management of chronic diseases (58). Leveraging its powerful algorithms, AI can efficiently analyze the vast amounts of data generated by CGM devices, identify potential patterns and correlations, accurately predict future blood glucose trends, and even detect early signs of risk (59). This provides patients and their medical teams with highly personalized management strategies based on big data. Such intelligent analytical tools undoubtedly bring unprecedented scientific rigor and foresight to diabetes management.

The integration of continuous glucose monitoring CGM technology with AI has revolutionized diabetes management by providing real-time, personalized insights for prevention, intervention, and treatment. However, despite the significant advancements, this integration also presents a range of complex challenges. **Table 1** summarizes the major challenges encountered in CGM-AI integration and outlines corresponding proposed solutions. Firstly, data integration remains a prominent issue. To achieve comprehensive and refined management, it is crucial to effectively integrate CGM data with other health information about the patient, such as demographics, lifestyle habits, medical history, and treatment plans. Building a secure, reliable, and flexible data management system that ensures efficient integration, in-depth analysis, and accurate interpretation of data is a daunting task. Additionally, with the surge in the use of wearable devices for monitoring physical activity, integrating these devices seamlessly with CGM systems to achieve comprehensive data fusion is another pressing challenge.

**Table 1:** Major challenges and proposed solutions for integrating continuous glucose monitoring and artificial intelligence in prediabetes management.

Challenges	Proposed solutions
Data integration across devices and systems	Develop standardized interoperability protocols and adopt blockchain-secured data systems
Algorithm adaptability to diverse populations	Train models on multi-ethnic, multi-center datasets
Data privacy and security concerns	Implement blockchain technology and comply with GDPR and HIPAA standards
High costs of CGM devices and AI platforms	Develop affordable graphene-based sensors and subsidized public health programs

Addressing these challenges requires innovative solutions, including the integration of blockchain technology to ensure secure and transparent data management for AI-driven CGM systems. Blockchain operates as a decentralized, immutable ledger in which data transactions are encrypted, timestamped, and distributed across a network of nodes. This structure inherently prevents unauthorized data tampering and ensures traceability, providing an additional layer of security for sensitive health information collected from CGM devices. By integrating blockchain with AI-enabled CGM platforms, patient data can be securely stored and accessed while enabling real-time updates without compromising privacy.

Developing AI algorithms with high precision and strong adaptability also poses difficulties. These algorithms need to undergo deep learning and optimization based on a broad and diverse clinical data set to accurately predict blood glucose fluctuations, identify personalized risk factors, and provide practical management recommendations. Moreover, algorithm design must fully consider individual patient differences to ensure that each suggestion is accurately tailored to the patient’s actual needs. Blockchain facilitates the implementation of smart contracts based on predefined conditional automated data-sharing protocols. For example, patients could provide limited access to specific healthcare providers or researchers, ensuring that data utilization meets ethical and regulatory standards. This framework enhances patient autonomy and enables individuals to take control of their own health data while supporting collaborative research and innovation in diabetes management.

Apart from these technical challenges, issues such as regulatory compliance, privacy protection, and ethical considerations must not be overlooked. Blockchain technology supports interoperability by securely connecting CGM data with other electronic health records (EHRs) and wearable devices. This integration provides a holistic view of patient health, enabling AI algorithms to generate more accurate predictions and tailored interventions. Moreover, the transparency and auditability of blockchain ensure stakeholder accountability, addressing regulatory requirements such as the General Data Protection Regulation (GDPR) and the Health Insurance Portability and Accountability Act (HIPAA). Ai-driven CGM systems must comply with such regulations to ensure confidentiality of patient data and transparency of algorithmic decision-making. Blockchain technologies, through immutable ledgers and smart contracts, can enhance transparency, accountability, and patient consent management, aligning closely with GDPR principles. Additionally, incorporating explainable AI (XAI) techniques can significantly enhance transparency in decision-making processes, fostering patient and clinician trust.

Implementation of CGM and AI technology in low- and middle-income countries (LMICs) poses distinct challenges, primarily due to high device costs (ranging from $300 to $1,000 per sensor) and limited healthcare infrastructure. In many LMICs, healthcare expenditures on diabetes care remain disproportionately low; for instance, diabetes care accounts for less than 2% of healthcare spending in sub-Saharan Africa (60). Economic studies further highlight that current patient expenditure for diabetes care in countries like Nepal averages approximately $23 per half-year, demonstrating the stark contrast with the cost of CGM technologies (61). Infrastructure limitations such as intermittent internet connectivity and low smartphone penetration further complicate the deployment of these advanced technologies. However, cost-effective, adaptive solutions like SMS-based interventions or offline AI models show promising results in improving glycemic control in resource-limited settings. Innovations, such as the development of low-cost graphene-based CGM sensors, coupled with targeted public health initiatives and international collaborations like the WHO Global Diabetes Compact, may offer viable strategies to bridge these gaps (WHO Global Diabetes Compact, 2021). Addressing these economic and infrastructural barriers through innovation, subsidization, and workforce training will be critical for achieving widespread adoption of AI-driven CGM systems in LMICs.

Looking ahead, the deep integration of CGM and AI, combined with blockchain technology, presents vast potential in the field of diabetes management. Technological innovations will continue to drive CGM devices toward greater accuracy, convenience, and non-invasiveness, while AI algorithm optimization will further improve the accuracy of blood glucose predictions and the level of personalized management. Through interdisciplinary research and close collaboration, we can expect to see more intelligent closed-loop systems in the future that automatically adjust insulin doses based on real-time blood glucose data, as well as innovative applications integrating AI virtual assistants to provide personalized health guidance and psychological support around the clock. At the same time, integrating social determinants of health into the diabetes management system to achieve a comprehensive and multidimensional health management upgrade will also be an important direction for future development.

In summary, the fusion of blockchain and AI in CGM systems offers a transformative approach to diabetes management, ensuring data security, promoting trust, and enabling seamless data sharing. Future research should focus on optimizing the use of blockchain in healthcare, addressing scalability challenges, and validating its effectiveness through multicenter trials. To realize this vision, researchers, healthcare professionals, and policymakers must continue to collaborate, overcome technical challenges, and unlock the tremendous potential of these transformative technologies, ultimately bringing unprecedented benefits and hope to diabetes patients worldwide. To maximize the global impact of CGM-AI integration, addressing healthcare access disparities is imperative, notably in low- and middle-income countries (LMICs), which account for over 75% of diabetes-related deaths (62), in Sub-Saharan Africa, healthcare expenditure on diabetes accounts for less than 2% of total health budgets (60). The prohibitive cost of CGM devices remains a barrier in LMICs with constrained health budgets (63), necessitating innovation in affordable alternatives such as graphene-based biosensors (64). Meanwhile, infrastructure barriers—including intermittent internet connectivity and low smartphone penetration—demand adaptive solutions like SMS-based glucose alerts or lightweight AI models optimized for offline use. A pilot study in rural India demonstrated that AI-driven SMS interventions improved glycemic control among prediabetic populations by 18% (65), validating the potential of low-resource adaptations. Multistakeholder collaborations, such as the WHO Global Diabetes Compact (66), must prioritize equitable technology distribution and localized training programs aligned with Sustainable Development Goals (67). By addressing cost, infrastructure, and workforce gaps, CGM-AI integration can transcend geographical and economic boundaries, delivering scalable solutions for prediabetes management in underserved regions.

Despite the transformative potential of CGM-AI integration, its widespread adoption remains hindered by critical barriers related to affordability and accessibility. In LMICs, where approximately three-quarters of the global diabetic population resides in these underdeveloped regions (68), the prohibitive costs of CGM devices ($300–$1000 per sensor) and AI-driven analytics platforms may limit equitable access. A cross-sectional study from the perspective of Nepalese diabetes patients revealed that the average healthcare resource cost for managing type 2 diabetes over six months was just $22.87 per patient, rendering advanced technologies unaffordable for most (61). A U.S. study found that CGM could reduce long-term medical costs by 15–20% through early intervention, achieving a cost-effectiveness ratio of approximately $33,000 per quality-adjusted life year (QALY) when sensors are used for 10 days, yet upfront investments remain prohibitive (69). Further economic evaluations reinforce the sustainability of CGM-AI interventions by demonstrating long-term reductions in healthcare utilization and complications. To address this, collaborative development of low-cost, modular devices and implementation of government-subsidized programs are required, as demonstrated by Thailand’s National Diabetes Prevention Initiative (70).

## 5 Summary

This article provides an in-depth analysis of the integration of CGM technology and AI in the management of prediabetes, exploring its practical value and future potential in diagnosis, management, and intervention. The real-time, dynamic data collection capabilities of CGM, combined with AI-driven analytical tools, have brought revolutionary changes to the personalized management of prediabetes. Despite challenges in data processing, algorithm development, and ethical compliance, the significant advantages demonstrated by this technological integration—such as improving blood glucose monitoring accuracy, optimizing treatment plans, and promoting patient empowerment—have paved the way for a new approach to diabetes management. Future research and practice should focus on the development of higher-precision CGM devices, optimized AI algorithms, and integrated management systems. Through interdisciplinary collaboration and policy support, the full potential of this technology integration can be unlocked, offering a new paradigm for global health management in prediabetes (71).

## Data Availability

The original contributions presented in the study are included in the article/supplementary material. Further inquiries can be directed to the corresponding authors.

## References

[1] Hsueh WA Orloski L Wyne K. Prediabetes: the importance of early identification and intervention. Postgrad Med. (2010) 122:129–43. doi: 10.3810/pgm.2010.07.2180 20675976

[2] E. The Lancet Diabetes. Prediabetes: much more than just a risk factor. Lancet Diabetes Endocrinol. (2025) 13:165. doi: 10.1016/S2213-8587(25)00034-8 39956119

[3] American Diabetes Association. Diagnosis and classification of diabetes: Standards of care in diabetes—2025. Diabetes Care. (2025) 48:S27–49. doi: 10.2337/dc25-S002 PMC11635041 39651986

[4] Sacks DB Arnold M Bakris GL Bruns DE Horvath AR Lernmark Å. Guidelines and recommendations for laboratory analysis in the diagnosis and management of diabetes mellitus. Diabetes Care. (2023) 46:e151–99. doi: 10.2337/dci23-0036 PMC10516260 37471273

[5] Shah VN Kerr D. What is a normal glucose value? Lancet Diabetes Endocrinol. (2025) 13:172–4. doi: 10.1016/S2213-8587(25)00023-3 39923788

[6] Khan MAB Hashim MJ King JK Govender RD Mustafa H Al Kaabi J. Epidemiology of type 2 diabetes - global burden of disease and forecasted trends. J Epidemiol Glob Health. (2020) 10:107–11. doi: 10.2991/jegh.k.191028.001 PMC7310804 32175717

[7] Rett K Gottwald-Hostalek U. Understanding prediabetes: definition, prevalence, burden and treatment options for an emerging disease. Curr Med Res Opin. (2019) 35:1529–34. doi: 10.1080/03007995.2019.1601455 30935247

[8] Khan RMM Chua ZJY Tan JC Yang Y Liao Z Zhao Y. From pre-diabetes to diabetes: diagnosis, treatments and translational research. Medicina (Kaunas). (2019) 55(9):546. doi: 10.3390/medicina55090546 31470636 PMC6780236

[9] GBD 2021 Diabetes Collaborators. Global, regional, and national burden of diabetes from 1990 to 2021, with projections of prevalence to 2050: a systematic analysis for the Global Burden of Disease Study 2021. Lancet. (2023) 402:203–34. doi: 10.1016/S0140-6736(23)01301-6 PMC10364581 37356446

[10] Galaviz KI Weber MB Suvada KB Gujral UP Wei J Merchant R. Interventions for reversing prediabetes: A systematic review and meta-analysis. Am J Prev Med. (2022) 62:614–25. doi: 10.1016/j.amepre.2021.10.020 PMC10420389 35151523

[11] Grams J Garvey WT. Weight loss and the prevention and treatment of type 2 diabetes using lifestyle therapy, pharmacotherapy, and bariatric surgery: mechanisms of action. Curr Obes Rep. (2015) 4:287–302. doi: 10.1007/s13679-015-0155-x 26627223

[12] Galiero R Caturano A Vetrano E Monda M Marfella R Sardu C. Precision medicine in type 2 diabetes mellitus: utility and limitations. Diabetes Metab Syndr Obes. (2023) 16:3669–89. doi: 10.2147/DMSO.S390752 PMC10658811 38028995

[13] Martín-Carro B Donate-Correa J Fernández-Villabrille S Martín-Vírgala J Panizo S Carrillo-López N. Experimental models to study diabetes mellitus and its complications: limitations and new opportunities. Int J Mol Sci. (2023) 24(12):10309. doi: 10.3390/ijms241210309 37373455 PMC10299511

[14] Chen M Yang J Zhou JH Hao YX Zhang J Youn CH. 5G-smart diabetes: toward personalized diabetes diagnosis with healthcare big data clouds. IEEE Commun Mag. (2018) 56:16–23. doi: 10.1109/MCOM.2018.1700788

[15] Veluvali A Dehghani Zahedani A Hosseinian A Aghaeepour N McLaughlin T Woodward M. Impact of digital health interventions on glycemic control and weight management. NPJ Digit Med. (2025) 8:20. doi: 10.1038/s41746-025-01430-7 39789102 PMC11717909

[16] Contreras I Vehi J. Artificial intelligence for diabetes management and decision support: literature review. J Med Internet Res. (2018) 20:e10775. doi: 10.2196/10775 29848472 PMC6000484

[17] Ellahham S. Artificial intelligence: the future for diabetes care. Am J Med. (2020) 133:895–900. doi: 10.1016/j.amjmed.2020.03.033 32325045

[18] Oliver N Reddy M Leelarathna L. Continuous glucose sensor accuracy: beyond the headline metric. Lancet Diabetes Endocrinol. (2024) 12:934–8. doi: 10.1016/S2213-8587(24)00245-6 39419044

[19] Buch V Varughese G Maruthappu M. Artificial intelligence in diabetes care. Diabetes Med. (2018) 35:495–7. doi: 10.1111/dme.2018.35.issue-4 29368355

[20] Woldaregay AZ Årsand E Walderhaug S Albers D Mamykina L Botsis T. Data-driven modeling and prediction of blood glucose dynamics: Machine learning applications in type 1 diabetes. Artif Intell Med. (2019) 98:109–34. doi: 10.1016/j.artmed.2019.07.007 31383477

[21] Liu X Zhang J. Continuous glucose monitoring: A transformative approach to the detection of prediabetes. J Multidiscip Healthc. (2024) 17:5513–9. doi: 10.2147/JMDH.S493128 PMC11590642 39600717

[22] Menke A Casagrande S Cowie CC. Contributions of A1c, fasting plasma glucose, and 2-hour plasma glucose to prediabetes prevalence: NHANES 2011-2014. Ann Epidemiol. (2018) 28:681–85.e2. doi: 10.1016/j.annepidem.2018.07.012 30122354

[23] Rooney MR Fang M Ogurtsova K Ozkan B Echouffo-Tcheugui JB Boyko EJ. Global prevalence of prediabetes. Diabetes Care. (2023) 46:1388–94. doi: 10.2337/dc22-2376 PMC10442190 37196350

[24] Brambilla P La Valle E Falbo R Limonta G Signorini S Cappellini F. Normal fasting plasma glucose and risk of type 2 diabetes. Diabetes Care. (2011) 34:1372–4. doi: 10.2337/dc10-2263 PMC3114342 21498787

[25] Shilo S Keshet A Rossman H Godneva A Talmor-Barkan Y Aviv Y. Continuous glucose monitoring and intrapersonal variability in fasting glucose. Nat Med. (2024) 30:1424–31. doi: 10.1038/s41591-024-02908-9 38589602

[26] Seidu S Kunutsor SK Ajjan RA Choudhary P. Efficacy and safety of continuous glucose monitoring and intermittently scanned continuous glucose monitoring in patients with type 2 diabetes: A systematic review and meta-analysis of interventional evidence. Diabetes Care. (2024) 47:169–79. doi: 10.2337/dc23-1520 38117991

[27] Nomura A Noguchi M Kometani M Furukawa K Yoneda T. Artificial intelligence in current diabetes management and prediction. Curr Diabetes Rep. (2021) 21:61. doi: 10.1007/s11892-021-01423-2 PMC8668843 34902070

[28] van Doorn W Foreman YD Schaper NC Savelberg H Koster A van der Kallen CJH. Machine learning-based glucose prediction with use of continuous glucose and physical activity monitoring data: The Maastricht Study. PLoS One. (2021) 16:e0253125. doi: 10.1371/journal.pone.0253125 34166426 PMC8224858

[29] Lebech Cichosz S Hasselstrøm Jensen M Schou Olesen S. Development and validation of a machine learning model to predict weekly risk of hypoglycemia in patients with type 1 diabetes based on continuous glucose monitoring. Diabetes Technol Ther. (2024) 26:457–66. doi: 10.1089/dia.2023.0532 38215207

[30] Oikonomou EK Khera R. Machine learning in precision diabetes care and cardiovascular risk prediction. Cardiovasc Diabetol. (2023) 22:259. doi: 10.1186/s12933-023-01985-3 37749579 PMC10521578

[31] Annuzzi G Apicella A Arpaia P Bozzetto L Criscuolo S De Benedetto E. Exploring nutritional influence on blood glucose forecasting for type 1 diabetes using explainable AI. IEEE J BioMed Health Inform. (2024) 28:3123–33. doi: 10.1109/JBHI.2023.3348334 38157465

[32] Woldaregay AZ Årsand E Botsis T Albers D Mamykina L Hartvigsen G. Data-driven blood glucose pattern classification and anomalies detection: machine-learning applications in type 1 diabetes. J Med Internet Res. (2019) 21:18. doi: 10.2196/11030 PMC6658321 31042157

[33] Herrero P Reddy M Georgiou P Oliver NS. Identifying continuous glucose monitoring data using machine learning. Diabetes Technol Ther. (2022) 24:403–8. doi: 10.1089/dia.2021.0498 35099288

[34] Gilliam LK Hirsch IB. Practical aspects of real-time continuous glucose monitoring. Diabetes Technol Ther. (2009) 11 Suppl 1:S75–82. doi: 10.1089/dia.2008.0135 19469681

[35] Hermanns N Ehrmann D Kulzer B Klinker L Haak T Schmitt A. Somatic and mental symptoms associated with dysglycaemia, diabetes-related complications and mental conditions in people with diabetes: Assessments in daily life using continuous glucose monitoring and ecological momentary assessment. Diabetes Obes Metab. (2025) 27:61–70. doi: 10.1111/dom.15983 39375863 PMC11618240

[36] Ben-Yacov O Godneva A Rein M Shilo S Kolobkov D Koren N. Personalized postprandial glucose response-targeting diet versus mediterranean diet for glycemic control in prediabetes. Diabetes Care. (2021) 44:1980–91. doi: 10.2337/dc21-0162 34301736

[37] Ben-Yacov O Godneva A Rein M Shilo S Lotan-Pompan M Weinberger A. Gut microbiome modulates the effects of a personalised postprandial-targeting (PPT) diet on cardiometabolic markers: a diet intervention in pre-diabetes. Gut. (2023) 72:1486–96. doi: 10.1136/gutjnl-2022-329201 PMC10359530 37137684

[38] Moser O Riddell MC Eckstein ML Adolfsson P Rabasa-Lhoret R van den Boom L. Glucose management for exercise using continuous glucose monitoring (CGM) and intermittently scanned CGM (isCGM) systems in type 1 diabetes: position statement of the European Association for the Study of Diabetes (EASD) and of the International Society for Pediatric and Adolescent Diabetes (ISPAD) endorsed by JDRF and supported by the American Diabetes Association (ADA). Diabetologia. (2020) 63:2501–20. doi: 10.1007/s00125-020-05263-9 33047169

[39] Liu S Ko QS Heng KQA Ngiam KY Feng M. Healthcare transformation in Singapore with artificial intelligence. Front Digit Health. (2020) 2:592121. doi: 10.3389/fdgth.2020.592121 34713061 PMC8521861

[40] Khodve GB Banerjee S. Artificial intelligence in efficient diabetes care. Curr Diabetes Rev. (2023) 19:e050922208561. doi: 10.2174/1573399819666220905163940 36065921

[41] Toschi E Wolpert H. Utility of continuous glucose monitoring in type 1 and type 2 diabetes. Endocrinol Metab Clin North Am. (2016) 45:895–904. doi: 10.1016/j.ecl.2016.06.003 27823610

[42] Parkin CG Graham C Smolskis J. Continuous glucose monitoring use in type 1 diabetes: longitudinal analysis demonstrates meaningful improvements in HbA1c and reductions in health care utilization. J Diabetes Sci Technol. (2017) 11:522–8. doi: 10.1177/1932296817693253 PMC5505435 28745091

[43] Cappon G Acciaroli G Vettoretti M Facchinetti A Sparacino G. Wearable continuous glucose monitoring sensors: A revolution in diabetes treatment. Electronics. (2017) 6:16. doi: 10.3390/electronics6030065

[44] Johnson ML Martens TW Criego AB Carlson AL Simonson GD Bergenstal RM. Utilizing the ambulatory glucose profile to standardize and implement continuous glucose monitoring in clinical practice. Diabetes Technol Ther. (2019) 21:S217–s225. doi: 10.1089/dia.2019.0034 31169432

[45] Choi A Choi SY Chung K Chung HS Song T Choi B. Development of a machine learning-based clinical decision support system to predict clinical deterioration in patients visiting the emergency department. Sci Rep. (2023) 13:8561. doi: 10.1038/s41598-023-35617-3 37237057 PMC10220080

[46] De La Brosse L Calmels P Camalon T Rehn M Soulé P Caleca N. Novel Ai-based algorithm to detect and reconstruct meal real time using Cgm data. Diabetes Technol Ther. (2022) 24:A115–5.

[47] Glasgow RE Kurz D King D Dickman JM Faber AJ Halterman E. Twelve-month outcomes of an Internet-based diabetes self-management support program. Patient Educ Couns. (2012) 87:81–92. doi: 10.1016/j.pec.2011.07.024 21924576 PMC3253192

[48] Healey E Kohane I. LLM-CGM: A benchmark for large language model-enabled querying of continuous glucose monitoring data for conversational diabetes management. Pac Symp Biocomput. (2025) 30:82–93. doi: 10.1142/9789819807024_0007 39670363

[49] Ajjan RA Seidu S Riveline JP. Perspective of continuous glucose monitoring-based interventions at the various stages of type 2 diabetes. Diabetes Ther. (2024) 15:1657–72. doi: 10.1007/s13300-024-01607-5 PMC11263446 38907936

[50] Monnier L Colette C Dunseath GJ Owens DR. The loss of postprandial glycemic control precedes stepwise deterioration of fasting with worsening diabetes. Diabetes Care. (2007) 30:263–9. doi: 10.2337/dc06-1612 17259492

[51] Cui M Baek SS Crespo RG Premalatha R. Internet of things-based cloud computing platform for analyzing the physical health condition. Technol Health Care. (2021) 29:1233–47. doi: 10.3233/THC-213003 34092673

[52] Elwyn G. Shared decision making: What is the work? Patient Educ Couns. (2021) 104:1591–5. doi: 10.1016/j.pec.2020.11.032 33353840

[53] Mubeen F Low Wang CC Al Maradni A Shivaswamy V Sadhu AR. Digital health and shared decision-making in diabetes care - A survey initiative in patients and clinicians. Endocr Pract. (2023) 29:538–45. doi: 10.1016/j.eprac.2023.04.012 37178788

[54] Guan Z Li H Liu R Cai C Liu Y Li J. Artificial intelligence in diabetes management: Advancements, opportunities, and challenges. Cell Rep Med. (2023) 4:101213. doi: 10.1016/j.xcrm.2023.101213 37788667 PMC10591058

[55] Kerr D Ahn D Waki K Wang J Breznen B Klonoff DC. Digital interventions for self-management of type 2 diabetes mellitus: systematic literature review and meta-analysis. J Med Internet Res. (2024) 26:e55757. doi: 10.2196/55757 39037772 PMC11301119

[56] Moschonis G Siopis G Jung J Eweka E Willems R Kwasnicka D. Effectiveness, reach, uptake, and feasibility of digital health interventions for adults with type 2 diabetes: a systematic review and meta-analysis of randomised controlled trials. Lancet Digit Health. (2023) 5:e125–43. doi: 10.1016/S2589-7500(22)00233-3 36828606

[57] Strain T Wijndaele K Dempsey PC Sharp SJ Pearce M Jeon J. Wearable-device-measured physical activity and future health risk. Nat Med. (2020) 26:1385–91. doi: 10.1038/s41591-020-1012-3 PMC7116559 32807930

[58] Subramanian M Wojtusciszyn A Favre L Boughorbel S Shan J Letaief KB. Precision medicine in the era of artificial intelligence: implications in chronic disease management. J Transl Med. (2020) 18:472. doi: 10.1186/s12967-020-02658-5 33298113 PMC7725219

[59] Shen Y Kleinberg S. Personalized blood glucose forecasting from limited CGM data using incrementally retrained LSTM. IEEE Trans BioMed Eng. (2025) 72(4):1266–77. doi: 10.1109/TBME.2024.3494732 PMC11999170 39514345

[60] Brown JB Ramaiya K Besançon S Rheeder P Tassou CM Mbanya JC. Use of medical services and medicines attributable to diabetes in Sub-Saharan Africa. PloS One. (2014) 9:e106716. doi: 10.1371/journal.pone.0106716 25216268 PMC4162573

[61] Dahal PK Rawal L Ademi Z Mahumud RA Paudel G Vandelanotte C. Estimating the health care expenditure to manage and care for type 2 diabetes in Nepal: A patient perspective. MDM Policy Pract. (2023) 8:23814683231216938. doi: 10.1177/23814683231216938 38107033 PMC10725113

[62] W.H. Organization. Global report on diabetes. (2016).

[63] Atun R Davies JI Gale EAM Bärnighausen T Beran D Kengne AP. Diabetes in sub-Saharan Africa: from clinical care to health policy. Lancet Diabetes Endocrinol. (2017) 5:622–67. doi: 10.1016/S2213-8587(17)30181-X 28688818

[64] Fenech-Salerno B Holicky M Yao CN Cass AEG Torrisi F. A sprayed graphene transistor platform for rapid and low-cost chemical sensing. Nanoscale. (2023) 15:3243–54. doi: 10.1039/D2NR05838C 36723120

[65] Peiris D Praveen D Mogulluru K Ameer MA Raghu A Li Q. SMARThealth India: A stepped-wedge, cluster randomised controlled trial of a community health worker managed mobile health intervention for people assessed at high cardiovascular disease risk in rural India. PloS One. (2019) 14:e0213708. doi: 10.1371/journal.pone.0213708 30913216 PMC6435227

[66] W.H. Organization. Global Diabetes Compact. World Health Organization (2021).

[67] Allen LN Pullar J Wickramasinghe KK Williams J Roberts N Mikkelsen B. Evaluation of research on interventions aligned to WHO ‘Best Buys’ for NCDs in low-income and lower-middle-income countries: a systematic review from 1990 to 2015. BMJ Glob Health. (2018) 3:e000535. doi: 10.1136/bmjgh-2017-000535 PMC5841523 29527342

[68] Shen J Kondal D Rubinstein A Irazola V Gutierrez L Miranda JJ. A multiethnic study of pre-diabetes and diabetes in LMIC. Glob Heart. (2016) 11:61–70. doi: 10.1016/j.gheart.2015.12.015 27102023

[69] Wan W Skandari MR Minc A Nathan AG Winn A Zarei P. Cost-effectiveness of continuous glucose monitoring for adults with type 1 diabetes compared with self-monitoring of blood glucose: the DIAMOND randomized trial. Diabetes Care. (2018) 41:1227–34. doi: 10.2337/dc17-1821 PMC5961392 29650803

[70] Sranacharoenpong K Hanning RM. Diabetes prevention education program for community health care workers in Thailand. J Community Health. (2012) 37:610–8. doi: 10.1007/s10900-011-9491-2 21971628

[71] Zhang J Zhang Z Zhang K Ge X Sun R Zhai X. Early detection of type 2 diabetes risk: limitations of current diagnostic criteria. Front Endocrinol (Lausanne). (2023) 14:1260623. doi: 10.3389/fendo.2023.1260623 PMC10665905 38027114

